# Polymer Matrix Incorporated with ZIF-8 for Application in Nonlinear Optics

**DOI:** 10.3390/nano10061036

**Published:** 2020-05-28

**Authors:** Yuri A. Mezenov, Nikita K. Kulachenkov, Andrei N. Yankin, Sergey S. Rzhevskiy, Pavel V. Alekseevskiy, Venera D. Gilemkhanova, Semyon V. Bachinin, Vyacheslav Dyachuk, Valentin A. Milichko

**Affiliations:** 1 Department of Physics and Engineering, ITMO University, 197101 St. Petersburg, Russia; iurii.mezenov@metalab.ifmo.ru (Y.A.M.); nikita.kulachenkov@metalab.ifmo.ru (N.K.K.); andrei.yankin@metalab.ifmo.ru (A.N.Y.); serg.rzhevskii@gmail.com (S.S.R.); pavel.alekseevskiy@metalab.ifmo.ru (P.V.A.); v.gilemkhanova@gmail.com (V.D.G.); semyonbachinin@gmail.com (S.V.B.); slava_d83@mail.ru (V.D.); 2 A.V. Zhirmunsky National Scientific Center of Marine Biology, Far Eastern Branch, Russian Academy of Sciences, 690041 Vladivostok, Russia; 3 Institut Jean Lamour, Université de Lorraine, UMR CNRS 7198, F-54011 Nancy, France

**Keywords:** metal–organic frameworks, polymers, composite, nonlinear optics, second-harmonic generation

## Abstract

Polymers with embedded metal–organic frameworks (MOFs) have been of interest in research for advanced applications in gas separation, catalysis and sensing due to their high porosity and chemical selectivity. In this study, we utilize specific MOFs with high thermal stability and non-centrosymmetric crystal structures (zeolitic imidazolate framework, ZIF-8) in order to give an example of MOF–polymer composite applications in nonlinear optics. The synthesized MOF-based polymethyl methacrylate (PMMA) composite (ZIF-8–PMMA) demonstrates the possibility of the visualization of near-infrared laser beams in the research lab. The resulting ZIF-8–PMMA composite is exposed to a laser under extreme conditions and exhibits enhanced operating limits, much higher than that of the widely used inorganic materials in optics. Overall, our findings support the utilization of MOFs for synthesis of functional composites for optical application.

## 1. Introduction

Metal–organic frameworks (MOFs) represent a new class of crystalline materials consisting of metal-containing nodes connected by multitopic organic linkers. MOFs stand out due to their unconventional hierarchical structure, high porosity and flexibility, which turn them into prospective materials for many applications in chemistry [1], biology [2], and physics [3]. The features of the crystal structure of specific MOFs, such as structural diversity [4], the lack of an inversion center (non-centrosymmetric) [5], and high resistance to heating [6] and humidity [7], open up new perspectives for using them as active materials for nonlinear optical devices [8,9,10]. Moreover, the fabrication of MOF thin films and the integration with polymers [11] can trigger the development of new chemical technology [12] for the fabrication of active optical elements compatible with industrial devices and optical metrology.

The fabrication of composite materials, integrating MOF micro crystals with polymeric binders, has recently been investigated in relation to their diverse chemical applications [11,13,14,15,16,17,18,19]. The resulting thin films and membranes, with a thickness of hundreds of nanometers to microns, exhibit functional properties for advanced gas storage and separation. Generally, MOF compounds such as ZIF-8, UiO-66, MIL-101, MIL-53, and HKUST-1 and others [11] are incorporated into thermoplastic polymer matrices such as poly(methyl methacrylate) (PMMA), polysulfone (PSF), polydimethylsiloxane (PDMS) or polyvinylidene difluoride (PVDF). There, the homogenous distribution of MOF microcrystals with unperturbed crystallinity can be achieved for MOF loadings ranging from 2.5 up to 87 mass. From a physical point of view, the latter allows one to successfully employ the specific physical properties of MOFs, such as the generation of high optical harmonics and structural resistance under extreme conditions, in nonlinear optical applications. Indeed, highly intense ultraviolet (UV) and infrared (IR) laser radiation can damage the structure of organic nonlinear optical materials, limiting device operation cycles. In this sense, MOFs can be considered as competitive materials for nonlinear optics.

Here, we report the synthesis of MOF-based PMMA composites that allow for the visualization of near-infrared laser radiation under extreme conditions in the research lab. This is possible due to the stable second-harmonic generation (SHG) of non-centrosymmetric MOFs dispersed inside the optically transparent polymer matrix under highly intense laser pumping. We confirm the reliability of the composite under such irradiative conditions and regimes, which cause damage to inorganic materials, such as gold and silicon. Our findings support this approach for the preparation of functional MOF-based PMMA composites and significantly contribute to the field of nonlinear optics.

## 2. Materials and Methods

As a model MOF with a non-centrosymmetric structure, we utilized the commercial compound ZIF-8 (Sigma-Aldrich, Ludwigshafen, Germany), a member of a widespread zeolitic imidazolate framework subclass of MOFs [20]. This compound consists of a Zn tetrahedra coordinated with 2-methylimidazolate linker units, which results in a zeolite-like **sod** topology. ZIF-8 demonstrates a strong intrinsic SHG signal due to its non-centrosymmetric cubic I-43m space group symmetry [21]. The value of the second order nonlinear optical coefficient for ZIF-8 is also relatively high, compared to commercial inorganic crystals such as potassium dihydrogen phosphate (KDP) and other non-centrosymmetric MOFs [9,21]. Moreover, ZIF-8 is transparent in the visible and near-infrared ranges and is remarkably stable, both thermally [22] and chemically. Indeed, ZIF-8 remains SHG-active upon heating up to 450 °C, when decomposition occurs [21], while structural deformations start at ~300 in air [22,23]. The average size of used crystals was optically estimated to be several hundred nm, allowing us to detect a SHG signal [21].

**Figure 1:**
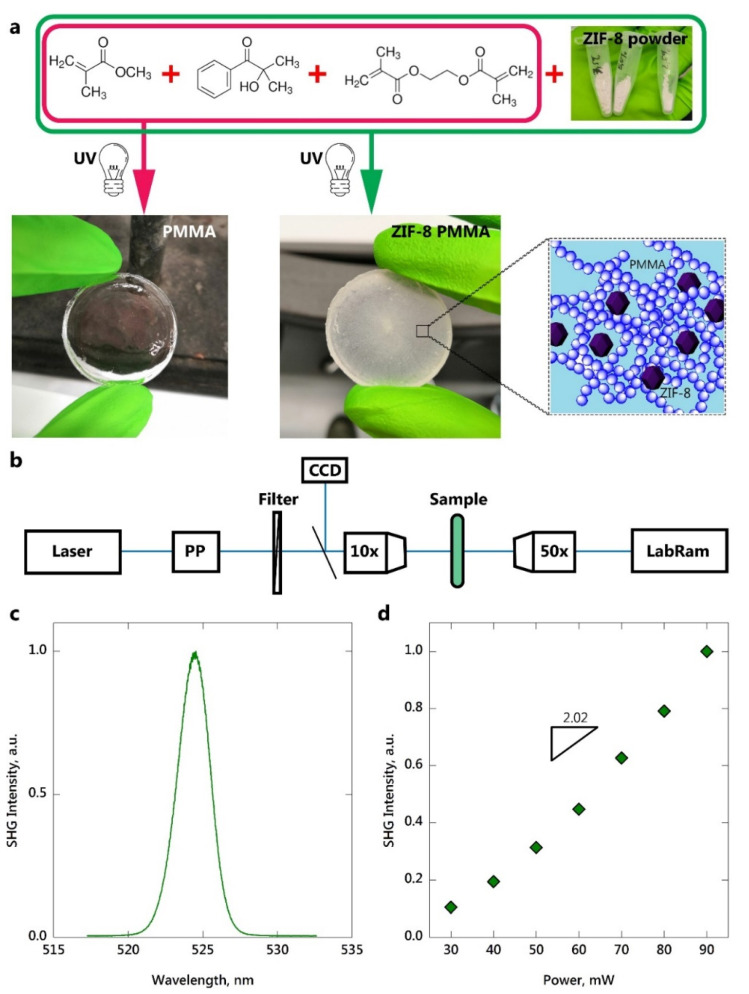
(**a**) Schematic illustration of ZIF-8– polymethyl methacrylate (PMMA) composite synthesis. (**b**) Optical scheme of imaging of the composite and analysis of SHG intensity. PP - pulse picker to produce 1 kHz pulse repetition rate; filter is used to tune the integral power. (**c**) Initial second-harmonic generation (SHG) signal of ZIF-8 powder excited by IR laser radiation (1047 nm central wavelength, 150 fs pulse duration, 50 mW, 80 MHz repetition rate) and corresponding SHG power dependence with a quadratic 2.02 ± 0.05 slope (**d**).

As a polymer, we selected PMMA, an optically transparent compound with a high resistance to heating of up to 150 °C [24]. For the synthesis of ZIF-8–PMMA composites, we used a solution based on methyl methacrylate (Sigma-Aldrich) as the main monomer, ethylene glycol dimethacrylate (Sigma-Aldrich) as a stitch reagent to create the 3D structure of polymethylmethacrylate, and 2-hydroxy-2-methylpropiophenone (Sigma-Aldrich) as a photoinitiator for UV polymerization in the ratio of 1:0.5:0.02, respectively. The powder weight of ZIF-8 was calculated as 4, 8 and 12 mass % depending on the weight of the monomer for each experiment. Before the process of polymerization under UV, a sufficient quantity of ZIF-8 was successfully dispersed in the main suspension within 30 s under sonication. The final suspension was exposed to UV irradiation for 10 min to create composites with different quantities of ZIF-8 in a matrix of polymethyl methacrylate. The resulting ZIF-8–PMMA composites have the form of pellets 1 inch in diameter and 2 mm in thickness (Figure 1a).

For the optical experiments, the pellets of ZIF-8 powder and ZIF-8–PMMA composite were placed on a glass substrate. The SHG spectra measurements in air were performed on a self-made confocal microscope setup (Figure 1b). The excitation was implemented via Mitutoyo M Plan APO NIR 10× objective (numerical aperture NA = 0.26) by Yb^3+^ laser (TeMa, Avesta Project, Troitsk, Russia) with a 1047 nm central wavelength and 150 fs pulse duration, operating at different regimes—an 80 MHz and a 1 kHz pulse repetition rates. The integral power of the laser varied from 0 to 500 mW (80 MHz) and 12 mW (1 kHz), allowing us to observe laser damaging (dewetting) or the ablation of inorganic solids like gold and silicon [25]. The transmission signal was collected by Mitutoyo HR NIR 50× objective (numerical aperture NA = 0.65) and then analyzed by a confocal spectrometer, HORIBA Labram (Longjumeau Cedex, France) with a water-cooling Andor DU 420A-OE 325 CCD (charge-coupled device) camera and 150-g/mm diffraction grating. The diameters of the area of excitation and the detection of the SHG signal were 5 and 1 μm, respectively. Optical images of the samples, irradiated by white light, and SHG signals from the pellets were obtained by a commercial CCD camera in reflection mode (Figure 1b).

In order to estimate the damage threshold of the ZIF-8–PMMA composite upon IR laser irradiation under different regimes, we compared the behavior of the composite with a 50-nm-thick Au film on the glass substrate and bulk crystalline silicon under similar extreme radiative conditions. For this purpose, the laser beam a with varied integral power and pulse repetition rate was focused on the surface of films and composites using a Mitutoyo M Plan APO NIR 10× objective (numerical aperture NA = 0.26); the imaging of the results of this light–matter interaction were performed by CCD (Figure 1b).

## 3. Results and Discussion

One of the key aspects of optical metrology in most research laboratories is measurement of laser beam parameters (wavelength, power and pulse energy, profile, coherence, etc.) and their adjustment. Most laser systems allow one to operate with coherent pulsed (ns, ps, and fs) and continuous wave (cw) radiation from the deep UV to IR regions. In this case, the optical elements of the optical systems can vary depending on the radiation parameters. Alignment systems, such as laser viewing cards [26], can be respectively universal, however. These cards are made of plastic with a photosensitive area. This area of each card makes it easy to determine the location of the UV, visible or IR laser beam and its focal point. However, the thermal effect of laser radiation of high intensity can lead to the destruction or burning of the surface layer. In this sense, utilizing PMMA embedded with the stable and non-centrosymmetric structures of MOFs can be crucial.

Figure 1a represents the resulting PMMA composites containing ZIF-8 microcrystals with different mass concentrations (4% to 12%). It should be noted that an increase in the mass fraction of ZIF-8 makes it possible to achieve a more uniform distribution of microcrystals within PMMA volume. Moreover, it allows for a more detailed visualization of the IR laser beam due to intense SHG signal and diffusion scattering between ZIF-8 microcrystals. The pellets of the composite were polished in order to form smooth surfaces and remove residual ZIF-8 powder.

Figure 1c,d represents SHG spectra for ZIF-8 powder. Since second-harmonic generation is a process of coherently emitting doubled frequencies (1/*λ*_1_ + 1/*λ*_1_ = 1/*λ*_2_), where *λ*_1_ and *λ*_2_ are the wavelengths of excitation (1047 nm) and emission, we detected an SHG signal at 523.5 nm. A quadratic slope (2.02 ± 0.05) for SHG intensity is also observed within the laser power, ranging from 30 to 90 mW (Figure 1d).

**Figure 2:**
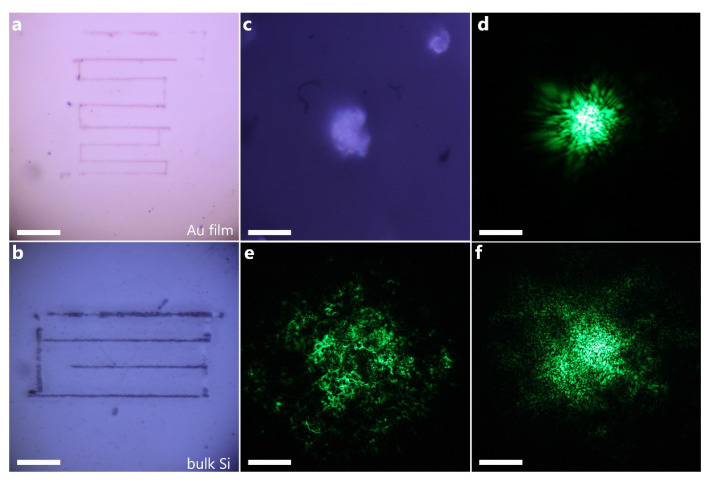
(**a**) Au film (50-nm thick) and (**b**) bulk crystalline silicon substrates damaged by laser radiation (1047 nm central wavelength, 150 fs pulse duration, 10 mW, 1 kHz repetition rate). Scale bars (**a** and **b**), 50 μm. Operation at 80 MHz pulse repetition rate results to more dramatic damaging of Au film and silicon. (**c**) Optical image of ZIF-8 powder with corresponding SHG signal (**d**) upon irradiation with an integral power of 100 mW (80 MHz). Scale bars (**c** and **d**), 20 μm. (**e**,**f**) Optical images of SHG for the ZIF-8–PMMA composite with 4 and 12% of ZIF-8. Scale bars (**e** and **f**), 50 μm. The optical images of SHG from ZIF-8–PMMA composite at 1 kHz pulse repetition rate are similar to (**e**,**f**).

In the second stage, we analyzed the ZIF-8–PMMA composite with 12 mass % of MOF crystals for the visualization of the laser beam with high intensity. The integral laser powers were chosen to be 10 and 100 mW for 1 kHz and 80 MHz pulse repetition rates, respectively. Under such extreme conditions, inorganic materials such as gold film (Figure 2a) and bulk silicon (Figure 2b) are completely destroyed. The laser beam causes the surface of gold to melt and makes silicon ablate due to high absorption coefficients [25]. This usually occurs at temperatures slightly lower than the melting point of the material. Such radiation conditions are also critical for organic polymers [27]. However, both the ZIF-8 powder and ZIF-8–PMMA composite demonstrate an intense SHG signal without visible damaging (Figure 2d–f). As mentioned above, ZIF-8–PMMA with a higher concentration of MOF crystals allows for a more detailed visualization of the laser beam profile (Figure 2f). Here, the more inhomogeneous the structure is, the more inhomogeneous the obtained SHG signal is (Figure 2e). Moreover, we irradiated ZIF-8 for 1 min by unfocused IR laser radiation with 500 mW integral power (80 MHz repetition rate), which corresponds to the power needed for damaging of several glasses and optical lenses [28]. Surprisingly, unchanged optical images of the microcrystals and SHG signal were detected.

**Figure 3:**
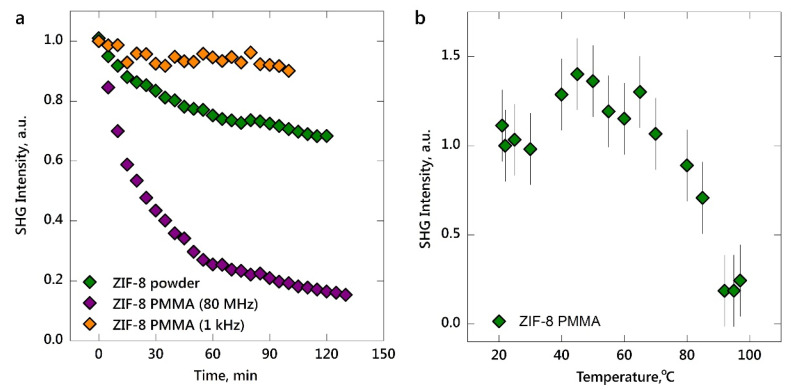
(**a**) Evolution of the SHG intensity for ZIF-8 powder and ZIF-8–PMMA composite upon laser radiation (1047 nm central wavelength, 150 fs pulse duration, 80 MHz and 1 kHz repetition rate) with an integral power of 100 mW (80 MHz) and 10 mW (1 kHz). (**b**) The thermal stability of SHG signal, excited by 50 mW at an 80 MHz repetition rate, for the ZIF-8–PMMA composite analyzed by heating on the Peltier element.

It is worth noting that decreasing the integral laser power (from 100 to 90 mW) reduces the brightness of the SHG picture by ~25%; however, it provides the significantly longer stability of the signal. Indeed, we have analyzed the operating time under extreme laser conditions—regimes of 100 mW, 80 MHz and 10 mW, 1 kHz. Figure 3a shows that the SHG intensity for a ZIF-8–PMMA composite irradiated with a 1 kHz repetition rate remains slightly constant (error of 10%) for 2 h. In this case, the heating effect of laser exposure does not affect the optical properties of the composite as it does for gold and silicon (Figure 2a,b). In contrast, upon irradiation by laser pulses with 80 MHz repetition rates, the SHG intensity decreases over time both for ZIF-8 powder and ZIF-8–PMMA composite. The intensity drops by 30% (for ZIF-8 powder) and 85% (for ZIF-8–PMMA) within 2 h of continuous exposure to the laser radiation. Further analysis of SHG signal intensity over time shows that the output of the signal reached a plateau. The behavior of ZIF-8 here can be explained by laser-induced heating: the inducing defects or thermodynamically possible rotation of linkers irreversibly create local centers of centrosymmetry [21,29]. Compared to ZIF-8 powder, the ZIF-8–PMMA composite demonstrates a higher decrease in SHG intensity. One possible explanation for such behavior is the laser-induced heating of the polymer matrix itself with the resultant damage to the irradiated area of the composite. Taking into account the fact that PMMA [24] demonstrates less thermal stability compared to ZIF-8 [22], the stronger decrease in the SHG signal for the composite can be attributed to the additional local deformations of the polymer during heating. This has also been confirmed by the analysis of the thermal stability of SHG intensity for the ZIF-8–PMMA composite (Figure 3b) during heating on the Peltier element. The decrease in the SHG intensity, excited by 50 mW at an 80 MHz repetition rate, starts at 80–90 °C. This temperature is more likely to affect the structure of the polymer with structural resistance to heating up to 150 °C [24] than MOF itself (300 °C [22,23], respectively).

## 4. Conclusions

In this study, we used specific MOFs (ZIF-8) with high thermal stability and non-centrosymmetric crystal structures to demonstrate of their applications in nonlinear optics. We synthesized an MOF-based polymer composite for the visualization of IR-pulsed laser radiation with different regimes (80 MHz and 1 kHz pulse repetition rates) and high integral power. The resulting ZIF-8–PMMA composite demonstrates a damage threshold much higher than that of widely used materials in optics, like gold and silicon. Herein, the composite demonstrates a more stable operation under the laser regime of 1 kHz for 2 h, while the laser regime of 80 MHz induces heating, with a negative effect on the optical properties of the composite. Overall, these findings support our approach to functional composite preparation and contribute to nonlinear optics research.
